# Prediction of survival after fetoscopic laser surgery for early‐onset twin‐to‐twin transfusion syndrome

**DOI:** 10.1002/uog.70178

**Published:** 2026-02-15

**Authors:** S. Prasad, F. G. Sileo, J. Binder, E. Brunelli, N. Chianchiano, C. M. Coutinho, F. D'Antonio, M. Döbert, A. Fichera, Y. Gielchinsky, K. Hecher, C. Iacovella, S. Malone, A. Martinez‐Varea, L. N. Nørgaard, C. Rodo, T. Simões, F. Slaghekke, Y. Yinon, A. Khalil, Collaborators, F. Bahlmann, E. Carreras, S. G. Alletti, O. Yaghi, E. Lopriore, M. M. Okido, A. Markovich, D. Mohammed, E. Moreno‐Perez, F. Prefumo, A. Queirós, J. Morales‐Roselló, K. Sundberg, M. Yeoh, A. Youssef, C. O. Ulusoy

**Affiliations:** ^1^ Fetal Medicine Unit, St George's Hospitals NHS Foundation Trust University of London London UK; ^2^ Prenatal Medicine Unit University of Modena and Reggio Emilia Modena Italy; ^3^ Department of Obstetrics and Feto‐Maternal Medicine Medical University of Vienna Vienna Austria; ^4^ Obstetric Unit, Department of Medical and Surgical Sciences University of Bologna and IRCCS Azienda Ospedaliero−Universitaria S. Orsola−Malpighi Bologna Italy; ^5^ Fetal Medicine Unit, Bucchieri La Ferla‐Fatebenefratelli Hospital Palermo Italy; ^6^ Hospital das Clínicas da Faculdade de Medicina de Ribeirão Preto, Universidade de São Paulo Ribeirão Preto Brazil; ^7^ Center for Fetal Care and High‐Risk Pregnancy, Department of Obstetrics and Gynecology University ‘G. d'Annunzio’ of Chieti−Pescara Chieti Italy; ^8^ Department of Obstetrics and Fetal Medicine University Medical Center Hamburg−Eppendorf Hamburg Germany; ^9^ Department of Clinical and Experimental Sciences University of Brescia Brescia Italy; ^10^ Fetal Medicine Center, Helen Schneider Hospital for Women, Rabin Medical Center Petach Tikvah Israel; ^11^ Faculty of Medical and Health Sciences Tel Aviv University Tel Aviv Israel; ^12^ Department of Obstetrics and Gynecology Buergerhospital − Dr. Senckenbergische Stiftung Frankfurt am Main Germany; ^13^ Department of Maternal−Fetal Medicine Royal Women's Hospital Melbourne Victoria Australia; ^14^ Department of Obstetrics and Gynecology La Fe University and Polytechnic Hospital Valencia Spain; ^15^ Center of Fetal Medicine, Department of Obstetrics Copenhagen University Hospital Rigshospitalet, Copenhagen Denmark; ^16^ Department of Obstetrics and Reproductive Medicine Maternal−Fetal Medicine Unit, Grup de Recerca en Medicina Materna i Fetal, Vall d'Hebron Institut de Recerca (VHIR), Vall d'Hebron Hospital Universitari Barcelona Spain; ^17^ Department of Maternal−Fetal Medicine, Alfredo da Costa Maternity Hospital Nova Medical School Lisbon Portugal; ^18^ Department of Obstetrics and Feto‐Maternal Medicine Leiden University Medical Center Leiden The Netherlands; ^19^ Fetal Medicine Unit, Department of Obstetrics and Gynecology, Sheba Medical Center, Faculty of Medical and Health Sciences Tel Aviv University Tel Aviv Israel; ^20^ Vascular Biology Research Centre Molecular and Clinical Sciences Research Institute, St George's University of London London UK; ^21^ Fetal Medicine Unit Liverpool Women's Hospital Liverpool UK

**Keywords:** early, intrauterine demise, laser, MCDA, monochorionic diamniotic, survival, TTTS, twin, twin‐to‐twin transfusion syndrome

## Abstract

**Objective:**

Data on early‐onset twin‐to‐twin transfusion syndrome (TTTS) are scarce and, therefore, evidence‐based counseling and management of these pregnancies are challenging. This study aimed to investigate survival rates and establish predictors of survival after fetoscopic laser surgery (FLS) for early‐onset TTTS.

**Methods:**

This was an international multicenter retrospective cohort study of monochorionic diamniotic twin pregnancies complicated by TTTS diagnosed before 18 + 0 weeks' gestation that underwent FLS. The primary outcome was dual‐twin survival at 28 days after birth. Secondary outcomes included survival of at least one twin and dual‐twin demise at 28 days after birth. Monoamniotic twin, triplet and higher‐order multiple pregnancies, pregnancies with chromosomal or structural fetal anomaly and TTTS cases not treated by FLS were excluded. Pre‐, intra‐ and postoperative characteristics were analyzed using multivariable logistic regression analysis. Discriminative performance was assessed using receiver‐operating‐characteristics‐curve analysis.

**Results:**

A total of 485 cases of early‐onset TTTS that underwent FLS were included. The rates of dual‐twin survival and survival of at least one twin at 28 days after birth were 51.5% (250/485) and 76.7% (372/485), respectively, while 23.3% (113/485) of cases resulted in dual‐twin demise. Multivariable logistic regression analysis showed that absent or reversed end‐diastolic flow (AREDF) in the donor umbilical artery (adjusted odds ratio (aOR), 0.487 (95% CI, 0.273–0.867)) and absent or reversed a‐wave in the donor ductus venosus (aOR, 0.299 (95% CI, 0.110–0.810)) at the time of TTTS diagnosis were associated independently with decreased odds of dual‐twin survival, while higher gestational age at birth was associated with increased odds of both dual‐twin survival (aOR, 1.172 (95% CI, 1.117–1.229)) and survival of at least one twin (aOR, 2.053 (95% CI, 1.699–2.481)). The model for dual‐twin survival showed modest discriminative performance with poor overall fit.

**Conclusions:**

The presence of AREDF in the donor umbilical artery and absent or reversed a‐wave in the donor ductus venosus, at the time of diagnosis of TTTS, and lower gestational age at birth were independent adverse predictors for dual‐twin survival following FLS in cases of TTTS diagnosed before 18 weeks. Future studies should explore the impact of surgical technique on survival rates. © 2026 The Author(s). *Ultrasound in Obstetrics & Gynecology* published by John Wiley & Sons Ltd on behalf of International Society of Ultrasound in Obstetrics and Gynecology.

## INTRODUCTION

Twin‐to‐twin transfusion syndrome (TTTS) affects up to 10–15% of monochorionic diamniotic (MCDA) twin pregnancies and is attributable largely to unbalanced arteriovenous anastomoses within the single shared placenta^1^. If left untreated and managed expectantly, TTTS is associated with a perinatal mortality rate of 80–90%^2^. Fetoscopic laser surgery (FLS) has been established as a standard of care for severe TTTS before 26 weeks' gestation following a landmark randomized controlled trial by the Eurofetus group^3^. Notably, the Eurofetus trial included only pregnancies at 15–26 weeks' gestation, with a median gestational age (GA) at randomization of 20.6 weeks. Similarly, the Quintero staging system for TTTS has been validated only for pregnancies from 18 weeks onwards^4^. Current international guidance advises fortnightly ultrasonographic surveillance of twin pregnancies for the development of monochorionicity‐specific complications, including TTTS, starting from 16 weeks^5^, ^6^, ^7^, ^8^. While most cases of TTTS present well after 16 weeks, there is a subset of cases that develop earlier and may require treatment before 18 weeks^9^. The published literature on the management and outcomes for early‐onset TTTS shows high perinatal mortality rates for all treatment modalities, but the available studies are generally of poor quality^10^. Parental counseling is based mostly on data from cases of TTTS diagnosed between 16 and 26 weeks. More robust data are needed to guide counseling about management options in the subset of cases with early‐onset TTTS. In the case of severe TTTS diagnosed very early in gestation, both FLS and selective termination may be considered by parents after appropriate counseling and informed consent, although the acceptability of these options is debatable among experts^11^, ^12^.

This study is part of the Early TTTS International Collaboration. Its aim was to determine survival rates and predictors of survival for both twins and at least one twin following FLS in MCDA twin pregnancies with TTTS diagnosed prior to 18 + 0 weeks. We hypothesized that certain pre‐, intra‐ and/or postoperative characteristics could influence survival outcome in MCDA twin pregnancies with early‐onset TTTS treated using FLS.

## METHODS

### Study design and population

This was an international multicenter retrospective cohort study comprising 17 tertiary fetal medicine referral centers that provide care to complex monochorionic twin pregnancies. A list of the participating centers is provided in Table S1. This study presents a focused analysis of a subgroup of MCDA twin pregnancies from participating centers for which early‐onset TTTS was diagnosed before 18 + 0 weeks and which underwent FLS between January 2007 and August 2023. The use of 2007 as the starting point was in recognition of the fact that the Eurofetus trial, which established FLS as the standard of care for severe TTTS before 26 weeks, was published in 2004^3^.

The inclusion criteria were MCDA twin pregnancies complicated by TTTS diagnosed before 18 + 0 weeks that subsequently underwent FLS. Exclusion criteria were triplet and higher‐order multiple pregnancies, monoamniotic twin pregnancies, MCDA twin pregnancies complicated by structural or chromosomal fetal anomaly and cases of TTTS managed by other modalities, namely expectant management, selective termination, termination of the entire pregnancy or amniodrainage.

GA was confirmed by the crown–rump length of the larger fetus at the first‐trimester scan or the date of oocyte retrieval or embryo transfer in spontaneous and assisted conceptions, respectively^13^. Monochorionicity was confirmed by an ultrasound scan performed at 11–14 weeks by demonstrating a thin intertwin membrane at the site of insertion of the amniotic membrane into the single placental mass (T‐sign)^5^. Patients underwent an ultrasound scan at least every 2 weeks from 16 weeks onwards. At each of these scans, the estimated fetal weight (EFW) and amniotic fluid deepest vertical pocket (DVP) were assessed for each fetus. Screening for aneuploidy, examination of detailed fetal anatomy and monitoring for monochorionicity‐related complications were performed as per local or international guidelines^5^.

The diagnosis of TTTS was made if there was polyhydramnios−oligohydramnios sequence, and classification was per the Quintero staging system^14^. Owing to the early GA, polyhydramnios was defined as a DVP ≥ 8 cm, while oligohydramnios was defined as a DVP ≤ 2 cm. In the absence of polyhydramnios, early‐onset TTTS was diagnosed by the presence of additional features of TTTS that do not fit the standard criteria. Specifically, these include: amniotic fluid discordance, defined as any obvious amniotic fluid difference not fulfilling the criteria for TTTS^15^; an absent bladder in the donor twin, along with a consistently distended bladder with rapid filling in the recipient twin^16^; or fetal cardiac dysfunction in at least one twin, consisting of cardiomegaly, cardiac hypertrophy, tricuspid regurgitation or reversed a‐wave in the ductus venosus on Doppler imaging^17^.

Selective fetal growth restriction (sFGR) was defined as either an EFW < 3^rd^ centile in one twin, or an EFW < 10^th^ centile in the smaller twin combined with an intertwin EFW discordance of ≥ 25%^5^. Intertwin EFW discordance was calculated using the formula: ((EFW of larger fetus − EFW of smaller fetus)/EFW of larger fetus) × 100 (%). The diagnosis of twin anemia–polycythemia sequence (TAPS) was made when there was evidence of intertwin discordance in the middle cerebral artery peak systolic velocity (MCA‐PSV) > 1 multiple of the median (MoM), or when the MCA‐PSV was > 1.5 MoM in the anemic twin and < 0.8 MoM in the polycythemic twin^18^, ^19^.

### Study outcomes and data collection

Data on maternal age, mode of conception, GA at diagnosis of TTTS, Quintero stage at diagnosis, concomitant sFGR and fetal Doppler parameters at diagnosis were ascertained from patient records. Umbilical artery Doppler abnormality was defined as persistent or intermittent absent or reversed end‐diastolic flow (AREDF).

In cases managed using FLS, as per the inclusion criteria, data were retrieved on bleeding, preterm prelabor rupture of membranes (PPROM) and fetal loss within 7 days post FLS, as well as on the recurrence of TTTS, the development of TAPS and/or the development of sFGR after 7 days post FLS. Perinatal outcomes, including live birth, intrauterine fetal demise (IUFD), neonatal death (NND) within 28 days after birth, perinatal death (defined as IUFD occurring beyond 22 weeks' gestation or NND), termination of pregnancy, GA at birth and birth weight, were documented.

Ethical and data governance procedures were followed at all participating centers in accordance with local regulations. The institutional review board at the coordinating center (St George's Hospital, London, UK) confirmed that formal ethical approval was not required for our retrospective analysis of routinely collected anonymous data.

The primary outcome measure was the rate of dual‐twin survival at 28 days after birth. Secondary outcome measures included the rate of survival of at least one twin and the rate of dual‐twin demise at 28 days after birth.

### Statistical analysis

Continuous variables were assessed for normality using the Shapiro–Wilk test and visual inspection of histograms and Q–Q plots. Continuous variables that followed a normal distribution are summarized as mean ± SD and were compared between groups using the independent‐samples *t*‐test. Continuous variables that were not normally distributed are reported as median (interquartile range (IQR)) and were compared between groups using the Mann–Whitney *U*‐test. Categorical variables are expressed as *n* (%), and between‐group comparisons were performed using the chi‐square test or Fisher's exact test, as appropriate.

Initially, we explored the differences in preoperative pregnancy and ultrasound findings, intraoperative features and immediate postoperative characteristics between pregnancies with and those without dual survival. *P*‐values were adjusted for multiple testing using the Benjamini–Hochberg procedure to control the false‐discovery rate, with a significance threshold of *q* = 0.05, and statistical significance was interpreted based on these adjusted values.

Subsequently, univariable and multivariable logistic regression analysis was performed to identify potential independent predictors of dual‐twin survival and survival of at least one twin. Model fit was evaluated using the Hosmer–Lemeshow goodness‐of‐fit test, and discriminative performance was assessed using the area under the receiver‐operating‐characteristics curve (AUC). Patients with incomplete data for explanatory variables were excluded from the analysis. Procedures such as checking for multicollinearity were included in standard post‐estimation diagnostics. Statistical analysis was conducted using SPSS version 24 (IBM Corp., Armonk, NY, USA) and RStudio version 2024.12.0+467 (Posit PBC, Boston, MA, USA). Statistical significance was defined as two‐sided *P* < 0.05.

## RESULTS

### Cohort selection and characteristics

**Figure 1:**
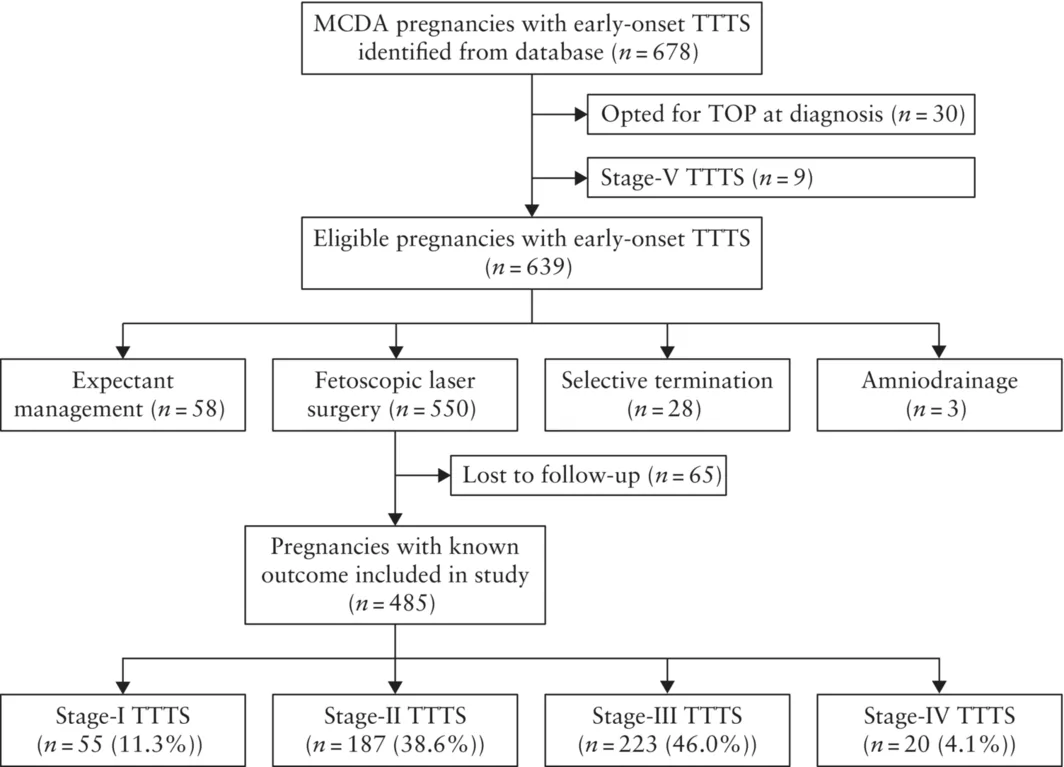
Flowchart summarizing inclusion in study of monochorionic diamniotic (MCDA) twin pregnancies that underwent fetoscopic laser surgery for early‐onset twin‐to‐twin transfusion syndrome (TTTS). TOP, termination of pregnancy.

During the study period, 678 MCDA twin pregnancies with early‐onset TTTS were identified from the electronic databases of the participating hospitals (Figure 1). Of those, 30 opted for termination of pregnancy at diagnosis and nine were diagnosed with Quintero Stage‐V TTTS, and were therefore excluded from further analysis. Of the remaining 639 cases, 58 were managed expectantly, three underwent amniodrainage only and 28 opted for selective termination, leaving 550 cases that were treated with FLS. After excluding 65 cases that were lost to follow‐up, the study cohort comprised 485 MCDA twin pregnancies that underwent FLS for early‐onset TTTS.

Table 1 presents the characteristics of the study cohort. The median GA at diagnosis of TTTS was 17.0 (IQR, 16.4–17.6) weeks. The distribution of TTTS severity based on the Quintero staging system showed a predominance of Stage III (46.0% (223/485)), followed by Stage II (38.6% (187/485)), Stage I (11.3% (55/485)) and Stage IV (4.1% (20/485)).

**Table 1:** Characteristics of 485 monochorionic diamniotic twin pregnancies with early‐onset twin‐to‐twin transfusion syndrome (TTTS) that underwent fetoscopic laser surgery (FLS)

Characteristic	Value (*n* = 485)
Maternal age (years)	31.2 ± 5.2
Mode of conception	
Spontaneous	388/440 (88.2)
ART	52/440 (11.8)
GA at diagnosis (weeks)	17.0 (16.4–17.6)
Quintero stage at diagnosis	
Stage I	55 (11.3)
Stage II	187 (38.6)
Stage III	223 (46.0)
Stage IV	20 (4.1)
Concomitant sFGR	130 (26.8)
Donor DVP at diagnosis (cm)	1.0 (0.0–1.7)
Recipient DVP at diagnosis (cm)	8.0 (7.0–9.0)
GA at FLS (weeks)	17.5 ± 1.0
Interval between diagnosis and FLS (days)	3.7 ± 6.1
Stage‐I TTTS*	9.5 ± 11.3
Stage‐II TTTS*	3.9 ± 5.6
Stage‐III TTTS*	2.5 ± 3.6
Stage IV TTTS*	0.0 ± 0.4
Preoperative cervical length (mm)	37.7 ± 8.2
Anterior placenta	207 (42.7)
Solomon technique	174/319 (54.5)
Post‐FLS complications†	177 (36.5)
PPROM	67 (13.8)
sFGR	46/410 (11.2)
TAPS	31/417 (7.4)
Recurrence of TTTS	21/419 (5.0)
GA at birth (weeks)	32.0 (27.0–35.0)
Donor birth weight (g)	1534 (1060–1909)
Recipient birth weight (g)	1770 (1308–2195)
Survival of at least one twin‡	372 (76.7)
Dual‐twin survival‡	250 (51.5)
Dual‐twin demise‡	113 (23.3)

Data are given as mean ± SD, *n*/*N* (%), median (interquartile range) or *n* (%).

* Quintero stage at diagnosis of TTTS.

† Including preterm prelabor rupture of membranes (PPROM), selective fetal growth restriction (sFGR), twin anemia−polycythemia sequence (TAPS), TTTS recurrence, bleeding and fetal loss.

‡ At 28 days after birth. ART, assisted reproductive technology; DVP, deepest vertical pocket; GA, gestational age.

The mean ± SD GA at FLS was 17.5 ± 1.0 weeks, with a mean cervical length at the time of surgery of 37.7 ± 8.2 mm. An anterior placenta was noted in 42.7% (207/485) of cases, and the Solomon technique of laser ablation was performed in 54.5% (174/319). Postoperative complications were recorded in 36.5% (177/485) of the cohort, with PPROM occurring in 13.8% (67/485) of cases. For the overall cohort, the rates of dual‐twin survival and survival of at least one twin at 28 days after birth were 51.5% (250/485) and 76.7% (372/485), respectively, while 23.3% (113/485) of cases resulted in dual‐twin demise. The mean interval between diagnosis of TTTS and FLS across the entire cohort was 3.7 ± 6.1 days. When stratified by Quintero stage at diagnosis, the mean interval was 9.5 ± 11.3 days for Stage I, 3.9 ± 5.6 days for Stage II, 2.5 ± 3.6 days for Stage III and 0.0 ± 0.4 days for Stage IV.

Table 2 presents preoperative pregnancy and sonographic characteristics, procedure‐related details and postoperative characteristics for pregnancies with two surviving twins and those with a single/no survivor. After adjusting for multiple comparisons using the Benjamini–Hochberg procedure, no significant differences were noted between the two groups in maternal age, GA at diagnosis, Quintero stage at diagnosis, concomitant sFGR, DVP of donor and recipient, Doppler characteristics of donor and recipient, preoperative cervical length and presence of an anterior placenta. There was also no significant difference in the mean GA at FLS between groups, nor in the use of the Solomon technique. The rate of postoperative PPROM was significantly higher in the single/no‐survivor group compared with the dual‐survivor group (19.6% *vs* 8.4%; *P* = 0.005). The single/no‐survivor group also had a significantly lower median GA at delivery (*P* = 0.005), with a higher frequency of delivery before 30 weeks (50.2% *vs* 20.8%; *P* = 0.005) and before 32 weeks (55.3% *vs* 37.6%; *P* = 0.005), compared with the dual‐survivor group.

**Table 2:** Pre‐, intra‐ and postoperative pregnancy and ultrasound characteristics in 485 monochorionic diamniotic twin pregnancies with early‐onset twin‐to‐twin transfusion syndrome that underwent fetoscopic laser surgery (FLS), according to survival status at 28 days after birth

Characteristic	Two survivors (*n* = 250)	One or no survivors (*n* = 235)	*P*
Maternal age (years)	31.1 ± 5.0	31.4 ± 5.5	0.660
GA at diagnosis (weeks)	17.0 (16.0–18.0)	17.1 (17.0–18.0)	0.899
Quintero stage at diagnosis			0.225
Stage I	36 (14.4)	19 (8.1)	
Stage II	99 (39.6)	88 (37.4)	
Stage III	107 (42.8)	116 (49.4)	
Stage IV	8 (3.2)	12 (5.1)	
Concomitant sFGR	57 (22.8)	73 (31.1)	0.120
Donor characteristics at diagnosis			
DVP (cm)	1.0 (0.0–1.5)	1.0 (0.0–2.0)	0.445
UA‐AREDF	68/219 (31.1)	86/207 (41.5)	0.084
Absent/reversed a‐wave in DV	8/175 (4.6)	19/163 (11.7)	0.071
Recipient characteristics at diagnosis			
DVP (cm)	8.0 (7.0–8.8)	8.0 (7.0–9.0)	0.628
UA‐AREDF	23/219 (10.5)	32/203 (15.8)	0.249
Absent/reversed a‐wave in DV	56/200 (28.0)	45/184 (24.5)	0.628
GA at FLS (weeks)	17.6 ± 1.0	17.4 ± 0.9	0.295
≤ 17 weeks	136 (54.4)	132 (56.2)	0.742
≤ 16 weeks	32 (12.8)	27 (11.5)	0.731
Preoperative cervical length (mm)	37.9 ± 7.6	37.5 ± 8.9	0.731
Anterior placenta	99 (39.6)	108 (46.0)	0.295
Solomon technique	98/176 (55.7)	76/143 (53.1)	0.731
PPROM post FLS	21 (8.4)	46 (19.6)	0.005
GA at birth (weeks)	33.0 (31.0–35.0)	27.0 (23.0–35.0)	0.005
< 30 weeks	52 (20.8)	118 (50.2)	0.005
< 32 weeks	94 (37.6)	130 (55.3)	0.005
< 34 weeks	169 (67.6)	145 (61.7)	0.304

Data are given as mean ± SD, median (interquartile range), *n* (%) or *n*/*N* (%). *P*‐values were adjusted using Benjamini–Hochberg false‐discovery rate correction (q = 0.05). AREDF, absent or reversed end‐diastolic flow; DV, ductus venosus; DVP, deepest vertical pocket; GA, gestational age; PPROM, preterm prelabor rupture of membranes; sFGR, selective fetal growth restriction; UA, umbilical artery.

### Predictors of dual survival and survival of at least one twin

On univariable analysis, higher Quintero stage at diagnosis (odds ratio (OR), 0.737 (95% CI, 0.578–0.940)), concomitant sFGR (OR, 0.655 (95% CI, 0.437–0.982)), AREDF in the donor umbilical artery at diagnosis (OR, 0.634 (95% CI, 0.426–0.943)), absent or reversed a‐wave in the donor ductus venosus at diagnosis (OR, 0.363 (95% CI, 0.154–0.854)) and postoperative PPROM (OR, 0.377 (95% CI, 0.217–0.654)) were associated with lower odds of dual survival at 28 days after birth (Table 3). In contrast, higher GA at birth was associated with higher odds of dual survival (OR, 1.156 (95% CI, 1.114–1.198)).

**Table 3:** Results of uni‐ and multivariable logistic regression analysis for prediction of dual‐twin survival at 28 days after birth in monochorionic diamniotic twin pregnancies with early‐onset twin‐to‐twin transfusion syndrome that underwent fetoscopic laser surgery (FLS)

Variable	OR (95% CI)	aOR (95% CI)
Quintero stage at diagnosis*	0.737 (0.578–0.940)	0.840 (0.371–1.900)
Concomitant sFGR*	0.655 (0.437–0.982)	0.698 (0.394–1.236)
AREDF in donor UA*	0.634 (0.426–0.943)	0.487 (0.273–0.867)
Absent/reversed a‐wave in donor DV*	0.363 (0.154–0.854)	0.299 (0.110–0.810)
AREDF in recipient UA	0.627 (0.353–1.113)	—
Absent/reversed a‐wave in recipient DV	1.201 (0.761–1.896)	—
Anterior placenta	0.771 (0.538–1.106)	—
Solomon technique	1.108 (0.711–1.725)	—
GA at diagnosis†	1.032 (0.843–1.263)	—
GA at FLS†	1.080 (0.910–1.282)	—
PPROM post FLS*	0.377 (0.217–0.654)	0.531 (0.252–1.119)
GA at birth*, †	1.156 (1.114–1.198)	1.172 (1.117–1.229)

* Variable included in multivariable model.

† Per 1‐week increase in predictor variable. aOR, adjusted odds ratio; AREDF, absent or reversed end‐diastolic flow; DV, ductus venosus; GA, gestational age; OR, odds ratio; PPROM, preterm prelabor rupture of membranes; sFGR, selective fetal growth restriction; UA, umbilical artery.

Multivariable logistic regression analysis demonstrated that AREDF in the donor umbilical artery (adjusted odds ratio (aOR), 0.487 (95% CI, 0.273–0.867)) and absent or reversed a‐wave in the donor ductus venosus (aOR, 0.299 (95% CI, 0.110–0.810)) at diagnosis were associated with decreased odds of dual survival, while higher GA at birth (aOR, 1.172 (95% CI, 1.117–1.229)) was associated with increased odds of dual survival (Table 3). The discriminative performance of the model was modest, with an AUC of 0.746 (95% CI, 0.687–0.804) (Figure S1), however the Hosmer–Lemeshow test indicated poor model calibration (*P* = 0.002) (Table S2).

Similarly, univariable analysis showed that higher Quintero stage at diagnosis (OR, 0.701 (95% CI, 0.522–0.940)) and postoperative PPROM (OR, 0.263 (95% CI, 0.154–0.450)) were associated with lower odds of survival of at least one twin (Table 4). Meanwhile, higher GA at FLS showed a trend towards increased odds of survival of at least one twin (OR, 1.247 (95% CI, 0.999–1.557)), and higher GA at birth was associated with higher odds of survival of at least one twin (OR, 2.093 (95% CI, 1.733–2.527)).

**Table 4:** Results of uni‐ and multivariable logistic regression analysis for prediction of survival of at least one twin at 28 days after birth in monochorionic diamniotic twin pregnancies with early‐onset twin‐to‐twin transfusion syndrome that underwent fetoscopic laser surgery (FLS)

Variable	OR (95% CI)	aOR (95% CI)
Quintero stage at diagnosis*	0.701 (0.522–0.940)	0.750 (0.043–12.953)
Concomitant sFGR	0.764 (0.482–1.213)	—
AREDF in donor UA	0.852 (0.537–1.353)	—
Absent/reversed a‐wave in donor DV	0.770 (0.312–1.898)	—
AREDF in recipient UA	0.613 (0.329–1.144)	—
Absent/reversed a‐wave in recipient DV	0.951 (0.552–1.639)	—
Anterior placenta	0.695 (0.456–1.061)	—
Solomon technique	1.213 (0.707–2.081)	—
GA at diagnosis†	1.217 (0.966–1.532)	—
GA at FLS*, †	1.247 (0.999–1.557)	1.099 (0.494–2.441)
PPROM post FLS*	0.263 (0.154–0.450)	0.397 (0.091–1.730)
GA at birth*, †	2.093 (1.733–2.527)	2.053 (1.699–2.481)

* Variable included in multivariable model.

† Per 1‐week increase in predictor variable. aOR, adjusted odds ratio; AREDF, absent or reversed end‐diastolic flow; DV, ductus venosus; GA, gestational age; OR, odds ratio; PPROM, preterm prelabor rupture of membranes; sFGR, selective fetal growth restriction; UA, umbilical artery.

On multivariable logistic regression analysis, only GA at birth was associated independently with higher odds of survival of at least one twin (aOR, 2.053 (95% CI, 1.699–2.481)) (Table 4). The model showed limited discriminative ability (AUC, 0.637 (95% CI, 0.578–0.697)) (Figure S2), and the Hosmer–Lemeshow test indicated poor model calibration (*P* = 0.002) (Table S2).

## DISCUSSION

### Summary of key findings

In this large multicenter cohort study, we report on the survival rates and predictors of survival after FLS for early‐onset TTTS diagnosed before 18 + 0 weeks' gestation. In our cohort, the rates of dual‐twin survival and survival of at least one twin at 28 days after birth were 51.5% and 76.7%, respectively, while 23.3% of cases resulted in dual‐twin demise. AREDF in the donor umbilical artery and absent or reversed a‐wave in the donor ductus venosus at the time of diagnosis of TTTS were independent adverse predictors of dual survival post FLS in cases of early‐onset TTTS. Higher GA at birth predicted increased odds of dual survival. The model demonstrated modest discriminative performance with poor overall fit.

### Interpretation of study findings and comparison with published literature

This is the largest cohort study to report data on MCDA twin pregnancies complicated by early‐onset TTTS and describe their outcomes following FLS. In our cohort, nearly half of the cases were Quintero Stage III at diagnosis. The higher incidence of Quintero Stage‐III TTTS among cases treated with FLS < 18 weeks and in the subset treated with FLS < 16 weeks has also been reported in a recent systematic review of Mustafa *et al*.^20^ and in a retrospective cohort study of Seaman *et al*.^21^. This may reflect the hesitancy of clinicians to intervene in early‐onset TTTS for a multitude of reasons, including the atypical appearance of the condition at these early gestations when a stringent polyhydramnios–oligohydramnios sequence may not be appreciable despite advanced functional impairment, in addition to the absence of clearly defined guidelines for both diagnosis and management^22^, ^23^. Clinicians may, therefore, adopt a policy of watchful waiting pending further evolution or progression of the condition^22^, ^23^. These challenges are compounded by added apprehension owing to the perceived risk of chorioamniotic membrane separation associated with fetal therapeutic procedures at these early gestations, when membranes have not yet, or have only recently, fused^22^, ^24^.

In our cohort, we report survival rates for both twins and at least one twin at 28 days after birth of 51.5% and 76.7%, respectively, following FLS for early‐onset TTTS. FLS for TTTS in conventional cohorts (16–26 weeks) has been associated with survival rates for both twins and at least one twin of up to 70% and 90%, respectively, highlighting the poorer outcome of early‐onset TTTS cases^2^, ^25^. Indeed, the severity and survival rate of early‐onset TTTS may be worse than that reported in the literature, as it is possible that many cases would have resulted in dual‐twin demise before even being recognized as TTTS, thus skewing the epidemiological data on the prevalence of the disease^26^, ^27^, ^28^.

In a cohort of 24 cases with early‐onset TTTS, Baud *et al*.^29^ reported survival rates for both twins and at least one twin of 56.5% and 83.8%, respectively, which are comparable with our findings. In our cohort, AREDF in the umbilical artery of the donor twin and absent or reversed a‐wave in the ductus venosus of the donor twin at diagnosis were associated independently with a lower dual‐survival rate. Concomitant sFGR and abnormal Doppler parameters of the donor fetus have been reported previously to be adverse prognostic predictors of survival in TTTS, even in cases with later‐onset TTTS^30^, ^31^, ^32^, ^33^.

There are conflicting reports of increased incidence of PPROM following FLS and its association with adverse perinatal outcome in TTTS. In our cohort, the overall rate of PPROM following FLS was 13.8% (67/485) and, on multivariable analysis, PPROM was not an independent predictor of dual survival following FLS. Baud *et al*.^29^ compared perinatal outcomes following FLS in ‘early’ (< 17 weeks), ‘late’ (> 26 weeks) and ‘conventional’ (17 − 26 weeks) TTTS cases. They reported a nearly 4‐fold higher rate of PPROM within 7 days after FLS in the early cohort (25.0%) compared with the late and conventional cohorts, despite similar pregnancy outcomes and survival rates across the early and conventional groups^29^. Their almost 2‐fold higher PPROM rate compared with the present cohort can be explained by the fact that the median GA at FLS in their cohort was 1 week earlier than that in our cohort. In our population, only 12.2% (59/485) underwent FLS ≤ 16 weeks. However, Espinoza *et al*.^34^ compared the outcome of TTTS cases treated using FLS before *vs* after 18 weeks and reported no significant difference in the incidence of PPROM or survival rates between the two groups. Contrary to our findings, Stirnemann *et al*.^35^ reported that FLS performed before 17 weeks significantly increased the risk of PPROM, with a 10% additional risk in the first week after surgery. While PPROM before 20 weeks was associated with a 56% risk of pregnancy loss in their cohort and, thus, worse survival rates, PPROM occurring after 20 weeks did not impact on survival, although it did lead to a higher incidence of preterm birth before 32 weeks. More recently, Brock *et al*.^36^ concluded that FLS < 18 weeks was associated with a lower rate of dual survival, and that the lower the GA at FLS, the higher the chance of PPROM. These conflicting reports may be attributed to variation in the GA cut‐offs for defining early TTTS and PPROM and for offering FLS, in addition to procedure‐related technical factors, including the choice of fetoscope, type of insertion (direct *vs* Seldinger), placental location, laser technique (Solomon *vs* selective) and, last but not least, the level of operator expertise, given the long learning curve associated with these procedures.

### Clinical and research implications

Early‐onset TTTS, presenting at a gestation remote from viability, is a challenging scenario for parental counseling owing to the limited availability of data regarding prognosis. The gold standard perinatal outcome following FLS would be remission of TTTS and dual‐twin survival, while avoiding iatrogenic preterm birth. Our findings support the tailoring of parental counseling regarding the prognosis for early‐onset TTTS cases, based on specific pre‐, intra‐ and postoperative characteristics. It would not be unreasonable to consider that, when acceptable and available, some parents might opt for selective termination, particularly in cases of severe TTTS at a very early GA^11^.

Our multicenter collaborative effort identified areas in which focused clinical and laboratory research is needed to optimize the perinatal outcome of these complicated pregnancies. Recently, a Delphi expert consensus concluded that FLS may be offered as early as 15 weeks in selected cases, and that clinicians should be guided by the severity of Doppler abnormalities^37^. Moreover, in the absence of an obvious polyhydramnios−oligohydramnios sequence, early cardiovascular markers of functional impairment or a modified diagnostic criterion for early‐onset TTTS may provide a more robust basis for offering fetal therapy in these cases and should be explored further^23^, ^38^, ^39^, ^40^.

With increasing operator experience, the burden of adverse perinatal outcome seems to have moved away from fatality and toward non‐fatal complications such as preterm labor and PPROM. The need for further research into the refinement of surgical techniques and adaptation of fetoscopic instruments for performing FLS at early GAs and according to placental location cannot be overemphasized. Feasibility studies have reported on the use of high‐intensity focused ultrasound as a non‐invasive method of selective placental vascular occlusion, and on the use of membrane‐anchoring devices in *ex‐vivo* and *in‐vivo* contexts to reduce the risk of PPROM^41^, ^42^, ^43^.

### Strengths and limitations

To our knowledge, this is the largest cohort study of early‐onset TTTS cases undergoing FLS reporting on survival rates and predictors of survival. All collaborating institutions are tertiary‐level fetal medicine institutions providing care for complicated MCDA twin pregnancies.

The main limitation of the study is the retrospective nature of the cohort, which carries an inherent risk of bias, and the exclusion of cases with incomplete outcome data, which incurs a risk of selection bias. Also, each unit followed its local protocols for the management of these complicated pregnancies. Moreover, with increasing experience in FLS, overall survival in TTTS has improved such that there is more interest in intact survival and long‐term neurocognitive outcome. These were not included as outcome measures in our study owing to a lack of sufficient data. In addition, prevailing uncertainty in the diagnostic criteria for early‐onset TTTS may have introduced some heterogeneity into case classification across centers. Furthermore, the definition of sFGR used in this study differs from the definitions currently in use, owing to the large timespan of the study. Multivariable models demonstrated only modest discriminatory ability, as reflected in the AUC values, and the Hosmer–Lemeshow test suggested that the models did not provide a good fit to the data. Possible explanations include residual data heterogeneity, presence of outliers and unmeasured confounding variables. Additionally, the models may not have captured potential interactions or non‐linear relationships between predictors. These factors may have limited the predictive performance of the models and should be considered when interpreting the results. Lastly, our analysis would have been stronger had more granular and consistent data been available on variables such as recurrent TTTS and post‐laser TAPS, to better assess their impact on survival outcome.

### Conclusions

In this large multicenter cohort of early‐onset TTTS cases diagnosed before 18 + 0 weeks undergoing FLS, about half (51.5%) had dual‐twin survival and 76.7% had at least one surviving twin at 28 days after birth. Lower GA at birth, AREDF in the donor umbilical artery and absent or reversed a‐wave in the donor ductus venosus at the time of diagnosis were independent adverse predictors for dual survival following FLS. Focused and concerted research is warranted to incorporate modified diagnostic criteria into clinical guidelines in order to aid timely diagnosis, referral and consensus‐led fetal therapy, and to adapt treatment techniques to early gestation to optimize perinatal outcome in these pregnancies.

### Collaborators

**F. Bahlmann**, Department of Obstetrics and Gynecology, Buergerhospital − Dr. Senckenbergische Stiftung, Frankfurt am Main, Germany

**E. Carreras**, Department of Obstetrics and Reproductive Medicine, Maternal−Fetal Medicine Unit, Grup de Recerca en Medicina Materna i Fetal, Vall d'Hebron Institut de Recerca (VHIR), Vall d'Hebron Hospital Universitari, Barcelona, Spain

**S. G. Alletti**, Fetal Medicine Unit, Bucchieri La Ferla‐Fatebenefratelli Hospital, Palermo, Italy

**O. Yaghi**, Fetal Medicine Unit, St George's Hospitals NHS Foundation Trust, University of London, London, UK

**E. Lopriore**, Department of Pediatrics, Leiden University Medical Center, Leiden, The Netherlands

**M. M. Okido**, Hospital das Clínicas da Faculdade de Medicina de Ribeirão Preto, Universidade de São Paulo, Ribeirão Preto, Brazil

**A. Markovich**, Fetal Medicine Unit, Department of Obstetrics and Gynecology, Sheba Medical Center, Faculty of Medical and Health Sciences, Tel Aviv University, Tel Aviv, Israel

**D. Mohammed**, Fetal Medicine Unit, St George's Hospitals NHS Foundation Trust, University of London, London, UK

**E. Moreno‐Perez**, Department of Obstetrics and Reproductive Medicine, Maternal−Fetal Medicine Unit, Grup de Recerca en Medicina Materna i Fetal, Vall d'Hebron Institut de Recerca (VHIR), Vall d'Hebron Hospital Universitari, Barcelona, Spain

**F. Prefumo**, UOC Ostetricia e Ginecologia, IRCCS Istituto Giannina Gaslini, Genova, Italy

**A. Queirós**, Department of Maternal−Fetal Medicine, Alfredo da Costa Maternity Hospital, Nova Medical School, Lisbon, Portugal

**J. Morales‐Roselló**, Department of Obstetrics and Gynecology, La Fe University and Polytechnic Hospital, Valencia, Spain

**K. Sundberg**, Center of Fetal Medicine, Department of Obstetrics, Copenhagen University Hospital, Rigshospitalet, Copenhagen, Denmark

**M. Yeoh**, Department of Maternal−Fetal Medicine, Royal Women's Hospital, Melbourne, Victoria, Australia

**A. Youssef**, Obstetric Unit, Department of Medical and Surgical Sciences, University of Bologna and IRCCS Azienda Ospedaliero−Universitaria S. Orsola−Malpighi, Bologna, Italy

**C. O. Ulusoy**, Ministry of Health, Perinatology Department, Etlik City Hospital, Ankara, Turkey

[Correction added on 8 October 2026, after first online publication: The collaborator name J. M. Rosello has been corrected to J. Morales‐Roselló in this version.]

## Supporting information


**Table S1** List of participating centers.
**Table S2** Predictive performance of models for survival following fetoscopic laser surgery for early‐onset twin‐to‐twin transfusion syndrome.

**Figure S1** Receiver‐operating‐characteristics curve for prediction of dual survival at 28 days after birth in cases of early‐onset twin‐to‐twin transfusion syndrome that underwent fetoscopic laser surgery.

**Figure S2** Receiver‐operating‐characteristics curve for prediction of survival of at least one twin at 28 days after birth in cases of early‐onset twin‐to‐twin transfusion syndrome that underwent fetoscopic laser surgery.

## Data Availability

The data that support the findings of this study are available from the corresponding author upon reasonable request.
