# Supplementary material for: Prediction of survival after fetoscopic laser surgery for early‐onset twin‐to‐twin transfusion syndrome
Source: Ultrasound Obstet Gynecol. 2026 Feb 15;67(3):295–303. doi: 10.1002/uog.70178 (PMC12951265; doi:10.1002/uog.70178)
Supplement: Supplementary file 1 — Table S1 List of participating centers. Table S2 Predictive performance of models for survival following fetoscopic laser surgery for early‐onset twin‐to‐twin transfusion syndrome. [file UOG-67-295-s001.docx]

**Table S1** List of participating centers

| **Participating Center** | **City and Country** |
| --- | --- |
| St. George’s Hospital | London, UK |
| Royal Women’s Hospital | Melbourne, Australia |
| Chaim Sheba Medical Center, Tel Hashomer Hospital | Tel Aviv, Israel |
| University of Chieti | Chieti, Italy |
| Helen Schneider Hospital for Women, Rabin Medical Center, Petach Tikvah, Israel; Faculty of Medicine, Tel Aviv University, Tel Aviv, Israel. | Petah Tikva, Israel |
| Maternidade Dr Alfredo da Costa | Lisbon, Portugal |
| Spedali Civili di Brescia | Brescia, Italy |
| Copenhagen University Hospital Rigshospitalet | Copenhagen, Denmark |
| Policlinico Sant’Orsola | Bologna, Italy |
| Medical University of Vienna | Vienna, Austria |
| Leiden University Medical Center | Leiden, the Netherlands |
| University Medical Center Hamburg-Eppendorf | Hamburg, Germany |
| Hospital Universitari Vall d’Hebron, Universitat Autonoma de Barcelona | Barcelona, Spain |
| Università degli studi di Palermo | Palermo, Italy |
| La Fe University and Polytechnic Hospital | Valencia, Spain |
| Department of Gynecology and Obstetrics, Hospital das Clínicas, Ribeirão Preto Medical School, University of São Paulo | Ribeirão Preto, Brazil. |
| Burgerhospital, Frankfurt am Main | Frankfurt |

**Table S2** Predictive performance of models for survival following fetoscopic laser surgery in early-onset twin-to-twin transfusion syndrome

|  | **AUROC** | **Std error** | **P value** | **95% CI** | **Goodness of Fit test (Hosmer and Lemeshow Test)** |
| --- | --- | --- | --- | --- | --- |
| Dual survivor | 0.746 | 0.030 | < 0.001 | 0.687-0.804 | P=0.002 |
| At least a single survivor | 0.637 | 0.031 | < 0.001 | 0.578-0.697 | P=0.002 |

AUROC, area under receiver-operating-characteristics curve.
