# Supplementary figures and images for: Prediction of survival after fetoscopic laser surgery for early‐onset twin‐to‐twin transfusion syndrome
Source: Ultrasound Obstet Gynecol. 2026 Feb 15;67(3):295–303. doi: 10.1002/uog.70178 (PMC12951265; doi:10.1002/uog.70178)

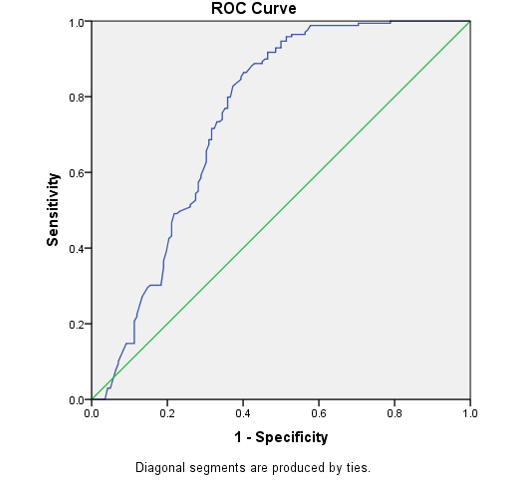

Supplement: Supplementary file 2 — Figure S1 Receiver‐operating‐characteristics curve for prediction of dual survival at 28 days after birth in cases of early‐onset twin‐to‐twin transfusion syndrome that underwent fetoscopic laser surgery. [file UOG-67-295-s002.png]

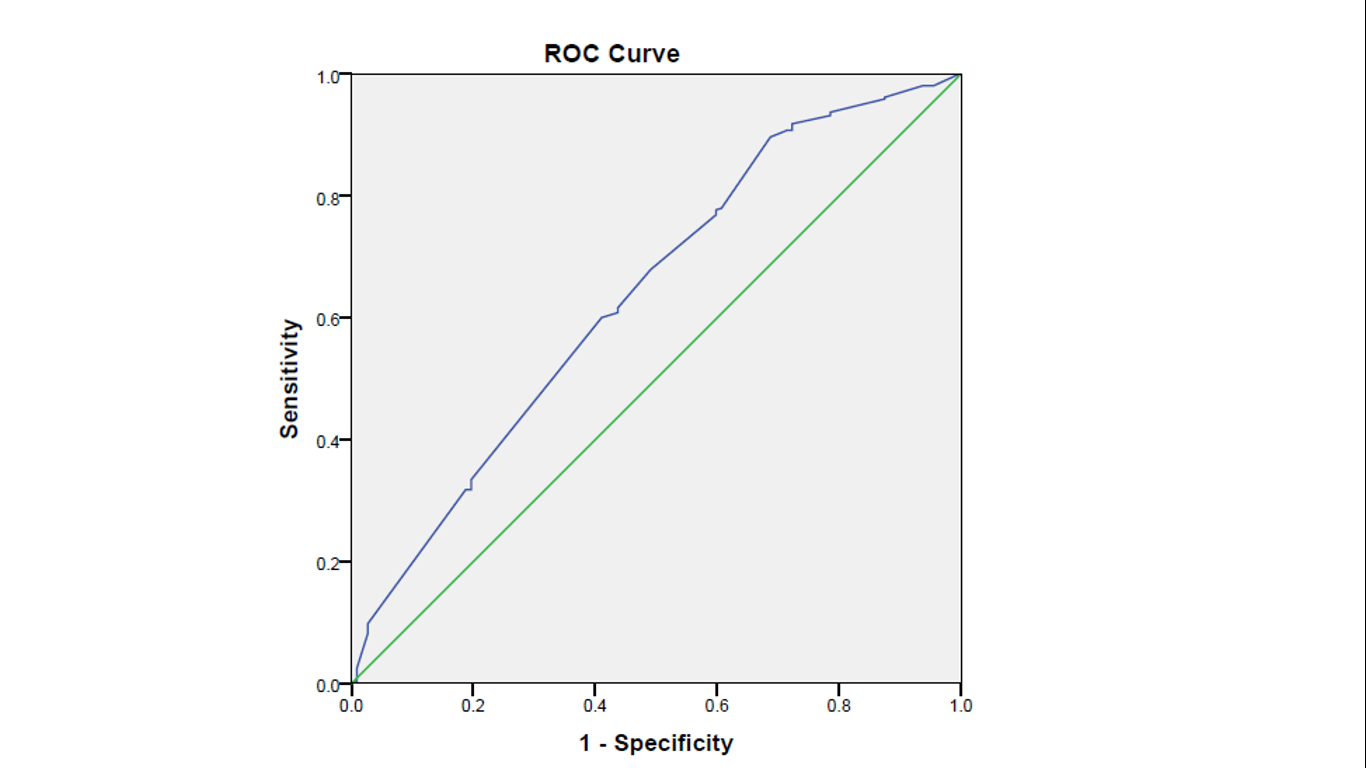

Supplement: Supplementary file 3 — Figure S2 Receiver‐operating‐characteristics curve for prediction of survival of at least one twin at 28 days after birth in cases of early‐onset twin‐to‐twin transfusion syndrome that underwent fetoscopic laser surgery. [file UOG-67-295-s003.png]
